# Special Issue: Genetics of Psychiatric Disease and the Basics of Neurobiology

**DOI:** 10.3390/genes13112008

**Published:** 2022-11-02

**Authors:** Laia Rodriguez-Revenga, Maria Isabel Alvarez-Mora

**Affiliations:** 1 Biochemistry and Molecular Genetics Department, Hospital Clinic of Barcelona, Institut d’Investigacions Biomèdiques August Pi i Sunyer (IDIBAPS), 08036 Barcelona, Spain; 2 CIBER of Rare Diseases (CIBERER), Instituto de Salud Carlos III, 28029 Madrid, Spain

A psychiatric disorder is a mental illness involving significant disturbances in thinking, emotional regulation or behavior. A large number of psychiatric disorders have been identified, and include depression, anxiety disorders, bipolar disorder, post-traumatic stress disorder (PTSD), schizophrenia, eating disorders, disruptive behavior, dissocial disorders and neurodevelopmental disorders. Overall, these conditions constitute a major public health burden, having high economic and social impacts worldwide. Many psychiatric disorders tend to run in families, suggesting potential genetic causes. In recent years, there has been an increasing number of genetic variants being detected to be associated with psychiatric disorders, following technological advances in molecular biology. High-throughput technologies, such as genome-wide association studies (GWASs), microarray analysis or next-generation sequencing (NGS), have allowed for the interrogation of genetic variation in thousands of individuals. Although there is still no complete genetic landscape of psychiatric disorders, promising findings have suggested that we are at the beginning of a novel era, with advanced efforts fostering the translation of this knowledge into a precise medical framework.

This Special Issue of *Genes* contains contributions from groups spread worldwide, reporting on state-of-the-art advancements in the area of genetics surrounding the basis of psychiatric diseases and neurobiology. It consists of seven original research articles, two reviews and one case report covering various aspects of different psychiatric disorders. Three original research publications focus on elucidating the functional effects of common gene variants in genes associated with psychiatric disorders. Ziegler and colleagues [1] in particular performed an association study on a functional intronic *CDH13* variant (rs2199430) with attention-deficit/hyperactivity disorder (ADHD). *CDH13* has been previously associated with a wide spectrum of neuropsychiatric disorders, including ADHD, autism and major depression. Although the rs22199430 genotype was not found to be associated with adult ADHD on the categorical diagnosis level, it was found to be associated with personality traits, suggesting an impact on neuronal processing when working on memory tasks [1]. On the other hand, Rafikova et al. investigated the contribution of 11 polymorphisms in 9 genes (*SLC6A4*, *HTR1A*, *HTR2A*, *HTR1B*, *SLC6A3*, *DRD4*, *DRD2*, *COMT* and *BDNF*) associating the serotonin and dopamine neurotransmitter systems as risk factors to the suicidal behavior and severity of symptoms of depression and anxiety in the Russian population. The authors proposed a variable number of tandem repeats (VNTR) in the *SLC6A3* gene (long and short alleles) as novel candidate biomarkers for suicidal behavior. They found the short allele to be more common in cases rather than controls, while carriers of homozygous long genotypes had a lower risk of suicide [2]. In addition, their results suggested that the LA allele of the *SLC64* gene (5-HTTLPR + rs25531) was more common in cases rather than controls, and that the rs4680 homozygous genotype in the *COMT* gene might play a protective role [2]. The work by Chmielowiec et al. [3] further investigated the association between rs4680 polymorphism in the *COMT* gene, and personality traits and anxiety in patients addicted to amphetamine. Using the standardized NEO five-factor inventory and state–trait anxiety inventory in patients dependent on amphetamine (compared to controls), results evidenced that the rs4680 homozygous genotype was statistically significantly associated with higher scores. Although the attempt to relate personality to consumer behavior should follow a multifactorial approach, the results reported in this study reinforced the idea that *COMT* may have an influential role in the risk of developing drug dependence.

In this Special Issue, two additional reports were published using high-throughput technologies. Concas et al. [4] performed a GWAS on temperament personality in 587 Italian individuals. The data analysis led to the identification of four new gens (*MAGI2*, *CALCB*, *BTBD3* and *PRKN*) associated with different temperament scales. Following a different approach, Rahman and coworkers [5] performed a meta-analysis of transcriptomic data derived from peripheral whole blood using the following keywords: “schizophrenia”, “blood” and “Homo sapiens”. They identified common processes between schizophrenia and the early onset of type 2 diabetes mellitus. The authors found inflammation-associated processes and membrane trafficking pathways to be common biological processes between both clinical conditions [5].

Fragile X syndrome (FXS) is the most common inherited cause of intellectual disability. It is caused by a CGG repeat expansion of the *FMR1* gene, which is also the most common single-gene cause of the autism spectrum disorder (ASD). Since FXS is inherited in an X-linked dominant pattern, males are usually more severely affected than females. For this reason, the neuropsychological profile in females is less described. Joga-Elvira et al. [6] performed a neuropsychological assessment on a group of girls with FXS, evidencing higher ratios of a lack of social support and communication, which could have led to them being placed in vulnerable situations.

With the increasing availability and implementation of NGS in clinical practice, Arteche-Lopez et al. [6] provided further data supporting the implementation of whole-exome sequencing (WES) as a first-tier test for the genetic diagnosis of ASD [7]. The authors provided genetic data from 343 ASD patients, evidencing the higher diagnostic power of WES compared to chromosomal microarray analysis and *FMR1* testing (WES, 75%; CMA, 20.4%; *FMR1* testing, 4.5%). In this context, Alvarez-Mora et al. [8] reported a case report of two siblings affected with a severe intellectual disability and other comorbidities, who embarked on a genetic testing odyssey until a diagnosis was reached through the use of whole-genome sequencing [8]. This case exemplified how advances in high-throughput technologies and their implementation worldwide have had considerable impact on the elucidation of the molecular causes underlying neurodevelopmental psychiatric disorders.

Finally, in their review, Lin and colleagues focused on recent studies of the cell recognition molecule neuroplastin (Np) and its gene (*Nptn/NPTN*) in relation to psychiatric and neurodegenerative diseases [9]. The recent identification of Np as a decisive component of plasma membrane Ca^2+^ ATPase complexes has opened the way to novel perspectives for the mechanistic understanding of learning and memory processes, and, thus, as a novel therapeutic target. In line with the hypothesis that disruptions to the immune system contribute to the development of schizophrenia, the review by Hogenaar and van Bokhoven addressed the question whether the classical pathway hyperactivity of the complement system contributes to the pathophysiology of schizophrenia through excessive synaptic pruning during postnatal development [10].

In summary, the current Special Issue “Genetics of Psychiatric Disease and the Basics of Neurobiology” covers various aspects of psychiatric disorder genetics, including rare and common genetic variants that might act as genetic risk factors. The herein collected data reinforce and support multiple novel biological hypotheses that could help unravel the genetic architecture of psychiatric disorders. This knowledge sets the stage for future research strategies with a potential translation to therapeutics.
